# Comparing the diagnostic accuracy of modified RIPASA and MASS in patients diagnosed with acute appendicitis in Suez Canal University Hospital Emergency Department: a cross-sectional study

**DOI:** 10.1186/s12873-022-00677-7

**Published:** 2022-08-08

**Authors:** Bassant Sayed Moussa, Mohamed Amin Ali, Dina AbdulRahman Ramadan Mohamed, Amal Mohamed El Shahhat

**Affiliations:** 1 Lecturer of Emergency Medicine, Faculty of Medicine, Suez Canal University, Ismailia, Egypt; 2 Resident of Emergency Medicine, Suez Canal Authority, Ismailia, Egypt; 3 Professor of Obstetric and Gynecology, Faculty of Medicine, Suez Canal University, Ismailia, Egypt

**Keywords:** Acute appendicitis, Sensitivity, Specificity, Negative appendicectomy rate, Predictive value, Accuracy

## Abstract

**Introduction:**

Acute appendicitis is the most common surgical condition presented in emergency departments globally. It is also the most common cause of abdominal pain treated surgically, with a lifetime risk of 7%. Recent studies show MASS to be easy, simple and cheap diagnostic tool for supporting the diagnosis of acute appendicitis.The modified RIPASA scoring system includes more parameters than MASS and the latter did not contain certain parameters. These parameters are shown to add to the accuracy of modified RIPASA over MASS especially in Asian population.

**Aim of this study:**

The aim of the study was to improve the diagnosis of acute appendicitis in order to lower the negative appendectomy rates.

**Patients & methods:**

This is cross sectional study, the study included 40 patients presented to the emergency department at Suez Canal University hospital with abdominal pain and suspected clinically as acute appendicitis. Then the decision of surgical intervention was made by surgeons, who were blinded for our study, based on their clinical judgment. Then both scores were calculated for all patients and other clinical data were obtained from patients after accepting being included in our study with an informed consent.After operations, the operating theatre records were obtained and cases pathological investigation of the appendices was done. Then the sensitivity, specificity, positive and negative predictive values were calculated and so the diagnostic accuracy for both scoring systems.

**Results:**

Clinically, all the patients were suffering acute right iliac fossa tenderness (100%), rebound tenderness (90%), and nausea/ vomiting (70%). Only 45% had elevated White blood count and 55% had negative urine analysis.

Histopathological analysis of appendices of the studied patients showed that 40% of the patients had suppurative appendicitis, one quarter of them had catarrhal appendicitis and only 20% had complicated perforated appendicitis. Meanwhile, about 15% had normal (negative) appendix**.** Modified RIPASA showed a good discriminative ability in our study where the area under the curve for modified RIPASA was 0.902 (95% CI: 0.798 – 1.00) (*p* = 0.002). Moreover, a value of 8.5 or higher was found to be the best cut-off point to predict acute appendicitis among patient suspected clinically as acute appendicitis with sensitivity = 70.6%, specificity = 100%, positive predictive value of 100%, and negative predictive value of 37.5% and 75% accuracy.The best cut-off score to diagnose acute appendicitis in our sample based on MASS was fixed at 5.5, where the sensitivity of the MASS reached 47.1%, with specificity of 33.3%, positive predictive value of 80%, negative predictive value of 10% and accuracy 45%.

**Conclusion:**

The modified RIPASA score is the best diagnostic scoring system for acute appendicitis if compared to the modified Alvarado score, with the former achieving significantly higher sensitivity and diagnostic accuracy. Modified RIPASA was concluded to be a more applicable and useful score. Negative appendicectomy rates can also be avoided by using modified RIPASA score.

**Supplementary Information:**

The online version contains supplementary material available at 10.1186/s12873-022-00677-7.

## Introduction

Acute appendicitis is the most common surgical condition presented in emergency departments globally. It is also the most common cause of abdominal pain treated surgically, with a lifetime risk of 7% [1].

The preoperative diagnosis of acute appendicitis is still a matter of difficulty especially in the extreme ages and females with gynecological or urinary problems, and depending mainly on a perfect and definite history and skillful clinical examination to avoid being exposed to negative appendectomy. Radiological studies can also help in the diagnosis of acute appendicitis including pelvi–abdominal ultrasound and Computerized Tomography (CT) scan, But the expensiveness and limited availability has limited its usage particularly in developing countries. So many scoring systems have been developed to help in diagnosis of acute appendicitis and lowering negative appendectomy rates [2].

Recent studies show Modified Alvarado Scoring Systems **(**MASS) to be easy, simple and cheap diagnostic tool for supporting the diagnosis of acute appendicitis. The scoring includes elements from the patient history, physical examination and laboratory tests [3]. This scoring system was proved to be of good sensitivity and specificity when used in Western populations [3].

The modified Modified Raja Isteri Pengiran Anak Saleha **(**RIPASA) scoring system includes more parameters than MASS and the latter did not contain certain parameters such as age, gender, duration of symptoms prior to presentation. These parameters are shown to add to the accuracy of modified RIPASA over MASS especially in Asian population [4].

### Aim of the study

aimed to compare both scores and calculate sensitivity, specificity, positive predictive value, negative predictive value and so diagnostic accuracy for both scores to know which score is more accurate for diagnosis of acute appendicitis.

### Hypothesis

Our study hypothesis is that modified RIPASA scoring system has a greater diagnostic accuracy compared to modified Alvarado scoring system (MASS) in patients presenting to emergency department with acute appendicitis.

### Objectives

The primary research objectives were (1) To determine the accuracy of modified RIPASA and MASS scoring systems in early diagnosis of acute appendicitis (2) to compare both scores so as to decrease the error in diagnosis and reduce the negative appendectomy rates.

The secondary objectives were: (1) To determine whether appendicitis has gender predominance. (2) To determine if appendicitis has age group predominance. (3) To estimate rates of negative appendectomy in Suez Canal university hospital.

### Patient and methods

The present study was designed as an observational cross-sectional study that included 40 patients presented to the emergency department at Suez Canal University hospital with abdominal pain and suspected clinically as acute appendicitis.

(b)inclusion criteria:—adults ( age more than 18 years old)—both genders—patients presented with acute abdominal pain and diagnosed by clinical data as acute appendicitis & undergoing surgical intervention.

(c)exclusion criteria:

- patients with other causes of acute abdomen other than acute appendicitis.
- patients transferred from our hospital after performing any medical or surgical intervention.
- pregnant females.

Sample size:

The sample size was calculated using the following formula [5]:

6 $$n={\left[\frac{{Z}_{\propto /2}+{Z}_{\beta }}{{P}_{1}-{P}_{2}}\right]}^{2} \left({p}_{1}{q}_{1} + {p}_{2}{q}_{2}\right)$$

where:

*n* = sample size.

Z α/2 = 2.576 (The critical value that divides the central 99% of the Z distribution from the 1% in the tail).

Z β = 1.28 (The critical value that separates the lower 10% of the Z distribution from the upper 90%).

P1 = Sensitivity in the MASS group = 63.3% [6].

P2 = Sensitivity in the RIPASA group = 96.2% (7).

So, by calculation, the sample size is equal to 40 subjects after the addition of the 10% drop-out proportion.

D)Material and Methods:

The study included patients who presented to emergency department with acute abdominal pain.

Data was collected in pre-organized data sheet by the researcher after approval from local ethical committee of Faculty of Medicine, Suez Canal University, and then all patients underwent the following:

Full medical history, Clinical evaluation of the patients was carried out on arrival to Emergency Department regarding:

Initial assessment:vital signs were recorded, then careful abdominal examination was done to identify the site of abdominal tenderness or rigidity, presence of rebound tenderness, rovsing sign. The condition of the patient either stable or unstable was assessed and accordingly the needed investigations and management were done. Then, Investigations:

Complete blood picture,• Urine analysis, Pregnancy test for females at reproductive age, Pelvi-abdominal ultrasound, CT abdomen if needed.

Treatment:Surgical team, who were blinded for our study, decided the plan of management and that patients needed surgical intervention.Then both scores, MASS and modified RIPASA scoring systems, were calculated for all patients after an informed written consent was taken from each patient without any obligation.

MASS is composed of 3 symptoms (migrating pain from the umbilicus to the right iliac fossa, anorexia, and vomiting), three signs (tenderness, rebound tenderness, and pyrexia) and one laboratory data (leukocytosis) yielding a total score of 9. It classifies patients into 4 groups of appendicitis probability (68):

• a) Unlikely diagnosis of appendicitis (1–4 points).
• b) Possible diagnosis (5-6points).
• c) Acute appendicitis present (7–8 points).
• d) Definitive diagnosis of acute appendicitis requiring surgery (9 points).

Modified RIPASA includes 14 clinical parameters (Gender, Age, right iliac fossa pain (RIF), pain migration to RIF, Anorexia, Nausea and vomiting, Duration of symptoms, RIF tenderness, Guarding, rebound tenderness, rovsing’s sign, Fever, Raised white cell count, negative urinalysis) with a total score of 16.5 (1).

• Probability of acute appendicitis is unlikely (< 5 points).
• Low probability of acute appendicitis (5–7 points).
• Probability of acute appendicitis is high (7.5- 11.5 points).
• Definitive diagnosis of acute appendicitis (12 points or more).

### Fate of the patient

After operation was done, operating theatre records were obtained and histopathological examination of the excised appendices was done.

### Expected outcomes

Improvement of the diagnosis of acute appendicitis in patients presenting to emergency department with acute abdominal pain.

## Results

The datasets generated and/or analysed during the current study are available in the [data sheet] repository uploaded as separate file.

This study included 40 patients with mean age of the patients was 30.10 ± 9.69 years with range from 18 to 50 years. Patients aged from 18 to 27 years formed 50% of the patients followed by (28 – 37) age group who formed 25% and (38 – 47) age group who formed (20%) as shown in (Table 1).

**Table 1:** Baseline characteristics of the study population (*n* = 40)

Variables	N (%)
**Age (years)**	
mean ± SD	30.10 ± 9.69
median (range)	28 (19 – 50)
**Age groups**	
18 – 27	20 (50)
28 – 37	10 (25)
38 – 47	8 (20)
48 – 57	2 (5)

Table 1 shows that the mean age of the patients was 30.10 ± 9.69 years with range from 19 to 50 years. Patients aged from 18 to 27 years formed 50% of the patients, followed by (28 – 37) age group who formed 25%, then patients aged (38–47) years old formed 20% and patients of (48 – 57) age group formed only (5%)

**Fig. 1:**
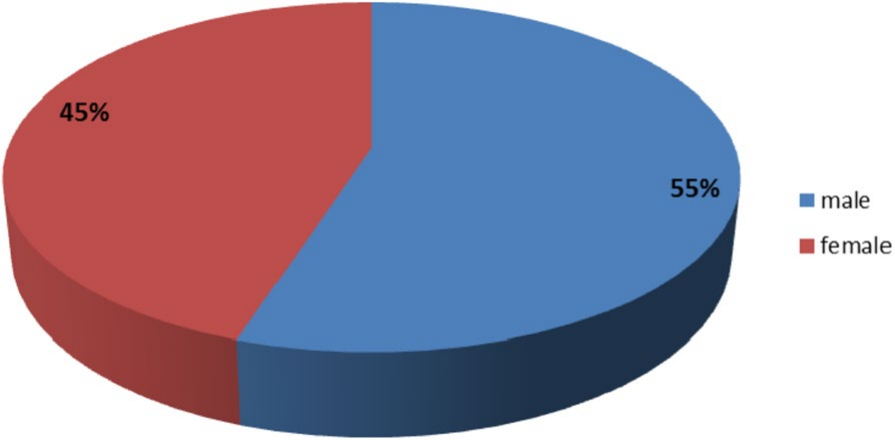
Distribution of gender among the studied participants. shows that male patients formed 55% of the sample, while females formed 45%

Our study showed male predominance, with 55% of male patients compared with 45% of female patients as shown in (Fig. 1).

Clinically, all the patients were suffering acute right iliac fossa tenderness, rebound tenderness (90%), and nausea/ vomiting (70%). Only 45% had elevated White blood count and 55% had negative urine analysis as shown in Table 2.

**Table 2:** Clinical and laboratory measures of the study sample (*n* = 40)

Variables	N (%)
**Manifestations**	
Rt. iliac fossa pain	18 (45)
Anorexia	16 (40)
Nausea and vomiting	28 (70)
Fever	10 (25)
Rt. iliac fossa tenderness	40 (100)
Guarding	22 (55)
Rebound tenderness	36 (90)
Rovsing sign	24 (60)
**Investigation**	
Elevated WBCs	18 (45)
Negative urine analysis	22 (55)

Table 2 summarizes clinical and laboratory characteristics of the studied sample. It was found that, among clinical manifestations presented by the patients, the top three presented manifestations were Rt. iliac fossa tenderness (100%), rebound tenderness (90%), and nausea/ vomiting (70%). Only 45% had elevated White blood count and 55% had negative urine analysis

**Fig. 2:**
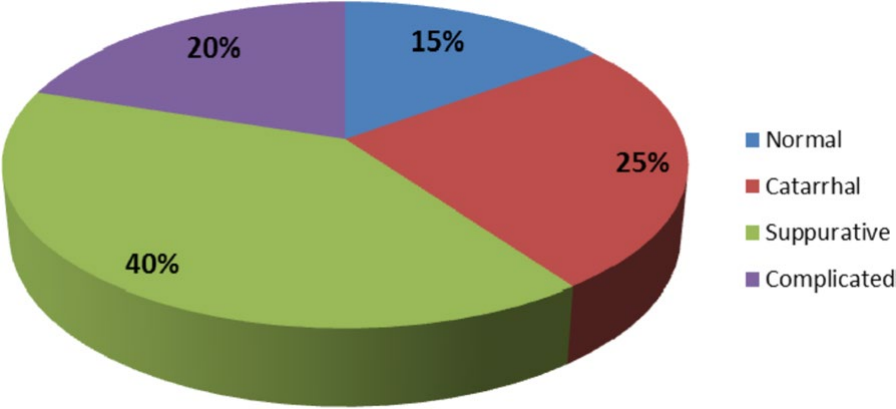
Histopathological analysis of appendices of the studied patients. Showed that 40% of the patients had suppurative appendicitis, one quarter of them had catarrhal appendicitis and only 20% had complicated perforated appendicitis. Meanwhile, about 15% had normal (negative) appendix

Histopathological analysis of appendices of the studied patients showed that 40% of the patients had suppurative appendicitis, one quarter of them had catarrhal appendicitis and only 20% had complicated perforated appendicitis. Meanwhile, about 15% had normal (negative) appendix as shown in (Fig. 2).

**Fig. 3:**
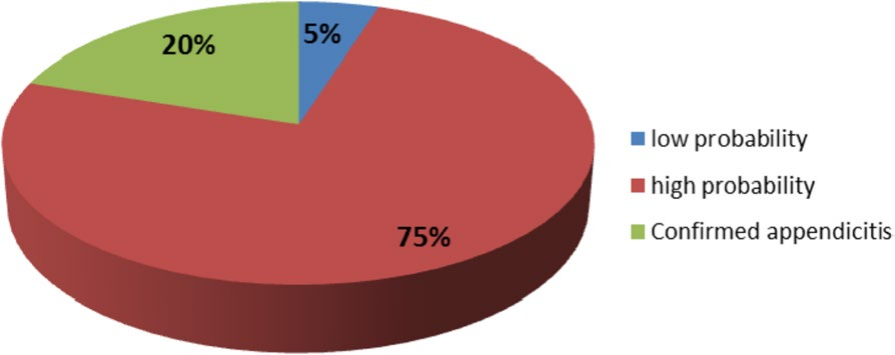
Likelihood of appendicitis diagnosis among patients based on modified RIPASA scoring system. shows that 75% of the patients had high probability of having appendicitis diagnosis and 20% had confirmed diagnosis based on modified RIPASA scoring system

The mean age of the patients with histopathological diagnosis was 27.8 ± 7.88 years with range from 19 to 44 years. Patients aged from 18 to 27 yrs formed 58.8% of the patients confirmed as acute appendicitis by histopathological diagnosis (34 patient) followed by (28 – 37) and (38–47) age groups where each formed (20.6%)as shown in Table 3.

**Table 3:** Age group distribution of patients with histopathological diagnosis (*n* = 34)

Variables	N (%)
**Age (years)**	
mean ± SD	27.8 ± 7.88
median (range)	27.8 (19 – 44)
**Age groups**	
18 – 27	20 (58.8)
28 – 37	7 (20.6)
38 – 47	7 (20.6)

Table 3 shows that the mean age of the patients with histopathological diagnosis was 27.8 ± 7.88 years with range from 19 to 44 years. Patients aged from 18 to 27 yrs formed 58.8% of the patients confirmed as acute appendicitis by histopathological diagnosis (34 patient) followed by (28 – 37) and (38–47) age groups where each formed (20.6%)

We found that acute appendicitis was significantly associated with younger age (*p* = 0.005), male gender (*p* = 0.003), nausea/ vomiting (*p* = 0.024) and guardening (*p* < 0.001) according to modified RIPASA diagnosis as shown in Table 4.

**Table 4:** Relationship between modified RIPASA diagnosis and baseline and clinical characteristics of the patients

Variables	Modified RIPASA score		*p*-value
	**Not appendicitis**	**Appendicitis**	
	**(*n* = 16)**	**(*n* = 24)**	
**Age (years)**, **mean ± SD**	35.13 ± 10.3	26.87 ± 7.6	0.005*^a^
**Gender**			
Male	4 (25)	18 (75)	0.003*^b^
Female	12 (75)	6 (25)	
**Manifestations**			
Rt. iliac fossa pain	16 (100)	20 (83.3)	0.136^c^
Anorexia	6 (37.5)	10 (41.7)	0.792^b^
Nausea and vomiting	8 (50)	20 (83.3)	0.024*^c^
Fever	2 (12.5)	8 (20)	0.163^c^
Rt. iliac fossa tenderness	14 (87.5)	24 (100)	0.154^c^
Guarding	2 (12.5)	20 (83.3)	< 0.001*^b^
Rebound tenderness	14 (87.5)	22 (91.7)	0.667^c^
Rovsing sign	10 (62.5)	14 (58.3)	0.792^b^
**Investigation**			
Elevated WBCs	6 (37.5)	12 (50)	0.52^b^
Negative urine analysis	6 (37.5)	16 (66.7)	0.10^b^

^a^ *p*-values are based on independent t-test. Statistical significance at *P* < 0.05

^b^ *p*-values are based on chi-square test. Statistical significance at *P* < 0.05

^c^ *p*-values are based on Fisher exact test. Statistical significance at *P* < 0.05

Table 4 shows the relationship between modified RIPASA diagnosis and baseline and clinical characteristics of the patients. It was found that acute appendicitis was significantly associated with younger age (*p* = 0.005), male gender (*p* = 0.003), nausea/ vomiting (*p* = 0.024) and guarding (*p* < 0.001) according to RIPASA diagnosis.

In this study patients had mean modified RIPASA of 9.70 ± 2.12 points with range from 7 to 15 points as shown in (Table 5). Also 75% of the patients had high probability of having appendicitis diagnosis and 20% had confirmed diagnosis based on modified RIPASA scoring system. Modified RIPASA showed a good discriminative ability in our study where the area under the curve for modified RIPASA was 0.902 (95% CI: 0.798 – 1.00) (*p* = 0.002). Moreover, a value of 8.5 or higher was found to be the best cut-off point to predict acute appendicitis among patient suspected clinically as acute appendicitis with sensitivity = 70.6%, specificity = 100%, positive predictive value of 100%, and negative predictive value of 37.5% and 75% accuracy as shown in (Tables 6 and 7).

**Table 5:** Modified Raja Isteri Pengiran Anak Saleha appendicitis scoring and modified Alvarado scoring system among acute appendicitis patients

Variables	N (%)
**Total RIPASA score**	
mean ± SD	9.70 ± 2.12
median (range)	9 (7 – 15)
**Total MASS**	
mean ± SD	5.60 ± 1.67
median (range)	5.5 (2 – 9)

Table 5 shows that the mean RIPASA among our sample was 9.70 ± 2.12 points with range from 7 to 15 points, while the mean MASS among our sample was 5.60 ± 1.67 points, with range from 2 to 9 points

**Table 6:** Area under the curve for modified RIPASA as a predictor of acute appendicitis

Variable	AUC	Stand. error	*p*-value	95% CI	Cut-off point	Sensitivity	Specificity
RIPASA	0.902	0.053	0.002*	(0.798 – 1.00)	8.5	70.6%	100%

Our study shows receiver operating characteristic curves for modified RIPASA as a predictor of acute appendicitis. Modified RIPASA showed a good discriminative ability where the area under the curve for modified RIPASA was 0.902 (95% CI: 0.798 – 1.00) (*p* = 0.002). Moreover, a value of 8.5 or higher was found to be the best cut-off point to predict acute appendicitis among patient with suspected clinically acute appendicitis with sensitivity = 70.6% and specificity = 100%

**Table 7:** Diagnostic predictive values of modified RIPASA and MASS for appendicitis against histopathological diagnosis

**Variables**	**Histopathological diagnosis**		**Diagnostic predictive values**				
	**Appendicitis** **(*n* = 34)**	**Not Appendicitis** **(*n* = 6)**	sensitivity	specificity	PPV	NPV	Accuracy
**RIPASA**							
appendicitis	24 (70.6%)	0 (0%)	70.6%	100%	100%	37.5%	
Not appendicitis	10 (29.4%)	6 (100%)					75%
**MASS**							
appendicitis	16 (47.1)	4 (66.7)	47.1%	33.3%	80%	10%	
Not appendicitis	18 (52.9)	2 (33.3)					45%

*PPV* Positive predictive value

*NPV* Negative predictive value

Table 7 shows validity testing of modified RIPASA and MASS for appendicitis against the gold standard culture test. The best cut-off score to diagnose acute appendicitis in our sample based on modified RIPASA was fixed at 8.5, where the sensitivity of the modified RIPASA testing reached 70.6%, with specificity of 100%, positive predictive value of 100%, and negative predictive value of 37.5% and 75% accuracy.

Whereas for MASS, the best cut-off score to diagnose acute appendicitis in our sample was fixed at 5.5, where the sensitivity of the MASS reached 47.1%, with specificity of 33.3%, positive predictive value of 80%, negative predictive value of 10% and accuracy 45%

Our study found thatregarding the relationship between MASS diagnosis and baseline and clinical characteristics of the patients, It was found that acute appendicitis was significantly associated with anorexia (*p* = 0.022), nausea/ vomiting (*p* = 0.014), fever (*p* < 0.001) and elevated WBCs (*p* < 0.001) according to MASS diagnosis. S shown in Table 8. Also the study patiets had mean MASS among 5.60 ± 1.67 points, with range from 2 to 9 points as shown in (Table 5). Whereas 30% of the patients had high probability of having appendicitis diagnosis and only 5% had confirmed diagnosis based on MASS. The best cut-off score to diagnose acute appendicitis in our sample based on MASS was fixed at 5.5, where the sensitivity of the MASS reached 47.1%, with specificity of 33.3%, positive predictive value of 80%, negative predictive value of 10% and accuracy 45% as shown in (Tables 7 and 9).

**Table 8:** Relationship between MASS diagnosis and baseline and clinical characteristics of the patients

Variables	MASS		*p*-value
	**Not appendicitis**	**Appendicitis**	
	**(*n* = 20)**	**(*n* = 20)**	
**Age (years)**, **mean ± SD**	30.9 ± 9.6	29.2 ± 9.9	0.586^a^
**Gender**			
Male	14 (70)	8 (40)	0.057*^b^
Female	6 (30)	12 (60)	
**Manifestations**			
Rt. iliac fossa pain	20 (100)	16 (80)	0.106^c^
Anorexia	4 (20)	12 (60)	**0.022**^**b**^
Nausea and vomiting	10 (50)	18 (90)	0.014*^b^
Fever	0 (0)	10 (50)	< 0.001*^b^
Rt. iliac fossa tenderness	18 (90)	20 (100)	0.487^c^
Guarding	10 (50)	12 (60)	0.751^b^
Rebound tenderness	18 (90)	18 (90)	1.00^c^
Rovsing sign	14 (70)	10 (50)	0.333^b^
**Investigation**			
Elevated WBCs	0 (0)	18 (90)	< 0.001*^b^
Negative urine analysis	12 (60)	10 (50)	0.751^b^

^a^ *p*-values are based on independent t-test. Statistical significance at *P* < 0.05

^b^ *p*-values are based on chi-square test. Statistical significance at *P* < 0.05

^c^ *p*-values are based on Fisher exact test. Statistical significance at *P* < 0.05

Table 8 shows the relationship between MASS diagnosis and baseline and clinical characteristics of the patients. It was found that acute appendicitis was significantly associated with anorexia (*p* = 0.022), nausea/ vomiting (*p* = 0.014), fever (*p* < 0.001) and elevated WBCs (*p* < 0.001) according to MASS diagnosis.

**Table 9:** Area under the curve for MASS as a predictor of acute appendicitis

Variable	Area	Stand. error	*p*-value	95% CI	Cut-off point	Sensitivity	Specificity
MASS	0.324	0.095	0.173	(0.137 – 0.510)	5.5	47.1%	33.3%

Our study shows receiver operating characteristic curves for MASS as a predictor of acute appendicitis Table 5 The area under the curve for MASS was 0.324 (95% CI: 0.137 – 0.510) (p = 0.173). Moreover, a value of 5.5 or higher was found to be the best cut-off point to predict acute appendicitis among patient with suspected clinically acute appendicitis with sensitivity = 47.1% and specificity = 33.3%

Our study found a poor agreement between modified RIPASA score and modified Alvarado score in diagnosis of appendicitis in patients (K = 0.201) as shown in (Table 10).

**Table 10:** Degree of agreement between modified RIPASA score and modified Alvarado score in diagnosis of appendicitis in patients

Variables	Modified RIPASA score		Kappa agreement	*p*-value
	**Not appendicitis** **(*n* = 16)**	**Appendicitis** **(*n* = 24)**		
**MASS**				
Not appendicitis (*n* = 20)	10 (62.5)	10 (41.7)	0.201	0.197
Appendicitis (*n* = 20)	6 (37.5)	14 (58.3)		

values are based on Man-Whitney test. Statistical significance at *P* < 0.05

Table 10 shows that there is a poor agreement between modified Alvarado and modified RIPASA scoring systems in regard to the diagnosis of acute appendicitis (Kappa value = 0.201, *p* = 0.197). The agreement in diagnosing appendicitis was found in only 58.3%.

## Discussion

Appendicitis is sufficiently common and appendectomy is the most frequently performed abdominal operation. The incidence of appendicitis is 1.5–1.9/1000, and it is ∼1.4 times greater in men than in women [7].

The definitive diagnosis of acute appendicitis is only possible with histopathology results after appendectomy. However, the decision to perform surgery is based solely on clinical evaluation supported by laboratory data. Therefore, diagnostic errors are common, resulting in perforation. Ultrasonography and CT scan are used nowadays [2]. Nevertheless, ultrasonography cannot replace clinical evaluation due to false-negative rates and non-availability in many medical institutes, which forces many surgeons to depend on clinical evaluation [8]**.**

Various scoring systems have been developed to support the diagnosis of acute appendicitis [2]. The classic Alvarado score is one of them and included left shift of neutrophil maturation yielding a total score of 10. However, in 1994, Kalan *et al.* omitted this parameter and produced a modified score. The Modified Alvarado Score (MASS) has comparable sensitivity and specificity to the classic Alvarado score, which were observed if the scores were applied to various populations and clinical settings, usually with worse yield when applied outside the population in which they were originally created [9]**.**

RIPASA scoring system for acute appendicitis was developed at 2010. Since the presentation of the system, it has been studied in Eastern and Western populations. There was a large foreign labor workforce in Brunei Darussalam, who must pay for their medical treatment at RIPAS Hospital. For this reason, foreign nationals tend to present much later when the symptoms are more severe**. **So they added the parameter of foreign NRIC in the score in these countries. Similar results have been demonstrated with the exclusion of the foreign identity card parameter, thus modified RIPASA developed [10].

So, this study aimed at comparing the diagnostic accuracy of modified RIPASA and MASS in diagnosing patients with acute appendicitis at emergency department of Suez Canal University hospital.

The present study was designed as an observational cross-sectional study that included 40 patients presented to emergency department at Suez Canal University hospital with abdominal pain and suspected clinically as acute appendicitis.

Regarding the baseline characteristics of the study population, this study showed that the mean age of the patients was 30.10 ± 9.69 years with range from 19 to 50 years. Patients aged from 18 to 27 years formed 50% of the patients, followed by (28 – 37) age group who formed 25%, then patients aged (38-47) years old formed 20% and patients of (48 – 57) age group formed only (5%) as shown in Table 1.

Reddy et al. at 2020 prospectively evaluated RIPASA score in 100 patients. The highest incidence of acute appendicitis (38%) was observed in age group of 21-30 years [11].

Our study showed male predominance, with 55% of male patients compared with 45% of female patients as shown in fig. 1. This is in agreement with other study in Africa by Malik et al. that showed male predominance [7]**.** Similarly, Chong et al. study about the development of RIPASA score, also showed the same proportion of male predominance where male to female ratio was 1.4:1 [12].

Contrast finding was reported at a retrospective survey carried out at south India by Naveen et al. at 2013 which revealed higher prevalence of appendicitis in females (51.7%), compared to males (48.2 %) [13].

According to the Clinical and laboratory measures of the studied population of our study, all the patients presented with right iliac fossa tenderness (100%), rebound tenderness (90%), and nausea/ vomiting (70%). Regarding investigations, only 45% of studied patients had elevated White blood count and 55% had negative urine analysis as shown in Table 2.

Similarly, Reddy et al. study showed that all of the studied patients were suffering from acute right iliac fossa pain (100%) [11].

Regarding the histopathological analysis of appendices of the studied patients showed that 40% of the patients had suppurative appendicitis, one quarter of them had catarrhal appendicitis and only 20% had complicated perforated appendicitis. Meanwhile, about 15% had normal (negative) appendices as shown in fig. 2.

Reddy et al. study reported that on histopathology, 90 patients were proven appendicitis (out of 90 cases, 40 reported as acute appendicitis, 23 as peri-appendicitis, 25 as acute suppurative and 2 cases as gangrenous appendicitis) and 10 negative cases were reported as (all of them were reactive lymphoid hyperplasia) [11] .

Study by Park et al. at 2013 reported a negative appendectomy rate of 15% [14]. This may be due to late presentation or misdiagnosis.

Concerning the diagnostic accuracy of both MASS and modified RIPASA scores, in this studied patients had mean modified RIPASA of 9.70 ± 2.12 points with range from 7 to 15 points as shown in Table 5.

**Fig. 4:**
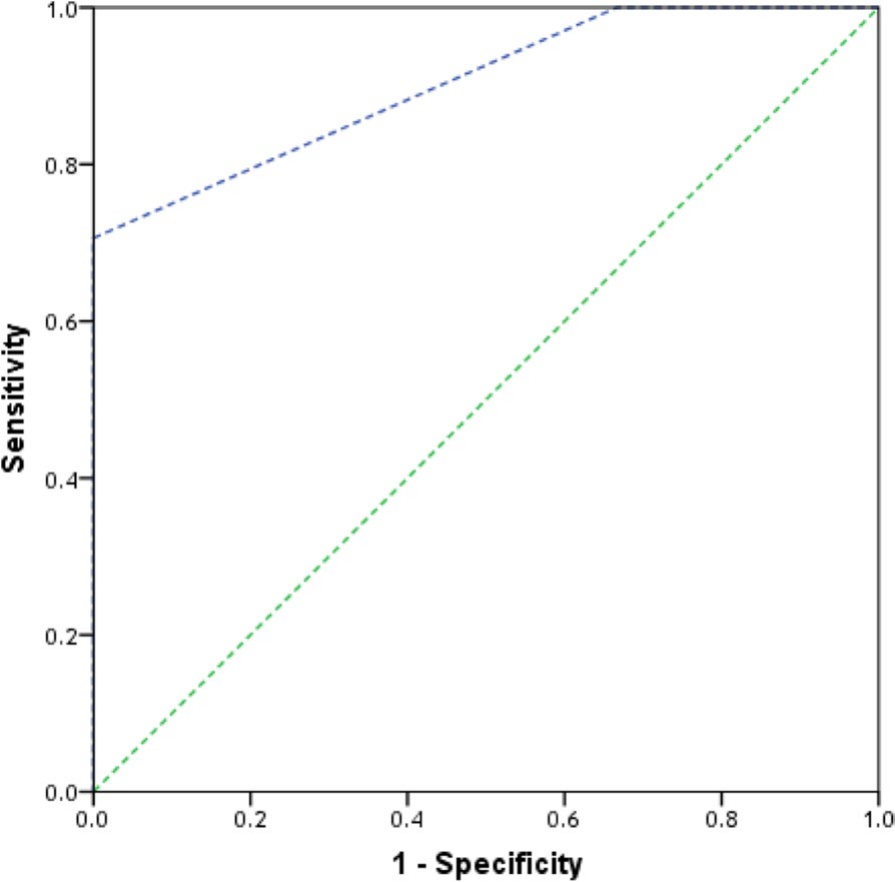
ROC curve of modified RIPASA for prediction of acute appendicitis. shows receiver operating characteristic curves for modified RIPASA as a predictor of acute appendicitis. Modified RIPASA showed a good discriminative ability where the area under the curve for modified RIPASA was 0.902 (95% CI: 0.798 – 1.00) (*p* = 0.002)

**Fig. 5:**
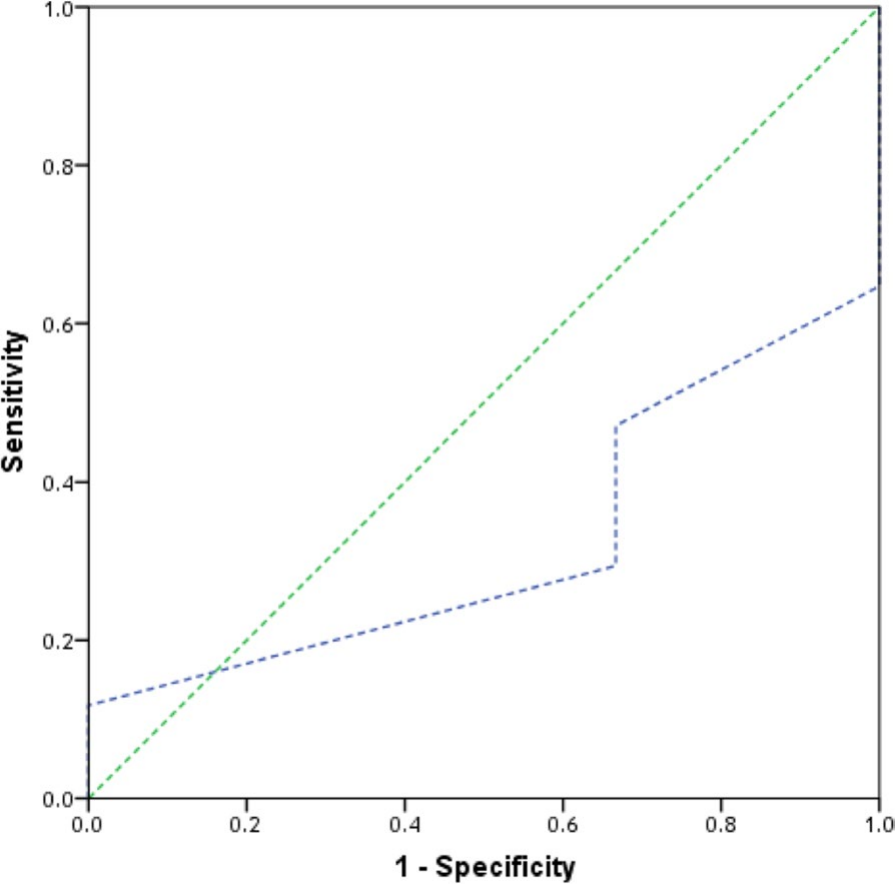
ROC curve of MASS for prediction of acute appendicitis. shows receiver operating characteristic curves for MASS as a predictor of acute appendicitis. The area under the curve for MASS was 0.324 (95% CI: 0.137 – 0.510) (*p* = 0.173)

. 75% of the patients had high probability of having appendicitis diagnosis and 20% had confirmed diagnosis based on modified RIPASA scoring system as shown in fig. 3. Modified RIPASA showed a good discriminative ability in our study where the area under the curve for modified RIPASA was 0.902 (95% CI: 0.798 – 1.00) (*p*=0.002). Moreover, a value of 8.5 or higher was found to be the best cut-off point to predict acute appendicitis among patient with clinically suspected acute appendicitis with sensitivity = 70.6%, specificity = 100% positive predictive value of 100 %, and negative predictive value of 37.5 % and 75% accuracy as shown in Table 6 and Fig. 4. While the area under the curve for MASS was 0.324 (95% CI: 0.137 – 0.510) (*p*=0.173). Moreover, a value of 5.5 or higher was found to be the best cut-off point to predict acute appendicitis among patient with clinically suspected acute appendicitis with sensitivity = 47.1% and specificity = 33.3%, positive predictive value of 80 %, negative predictive value of 10% and accuracy 45% as shown in Table 9 and Fig. 5 .

Our study found a poor agreement between modified RIPASA score and modified Alvarado score in diagnosis of appendicitis in patients (K=0.201) as shown in Table 10.

Reddy et al. study reported that on comparing both scores, Both p values were statistically significant. ROC curve shows a larger area under the curve for modified RIPASA when compared to MASS . This study also found that the cut-off score to diagnose acute appendicitis in modified RIPASA was fixed at 7.5 which yielded 90 % sensitivity and 72% specificity (which was higher for modified RIPASA score than MASS),positive predictive value 89% and NPV was 30% (the positive predictive value was higher for MASS and negative predictive value was higher for modified RIPASA score) [11].

Another study carried out at 2017 by Kumar et al. reported similar values, where sensitivity, specificity, PPV, NPV and accuracy of 84.2%,100%,100%,85% and 25% respectively [15].

Whereas Rathod et al. at 2015 reported 82.6% and 66.7% specificity and sensitivity respectively for modified RIPASA score [10].

Another study by Sammalkorpi et al. at 2014 found that a cut off value for positive MASS to be more than or equal to 7, showed better diagnostic parameters on these cut off values. When applied to patients, the MASS showed poor sensitivity, poor diagnostic accuracy, and good specificity (with a sensitivity of 59.6%, specificity of 87.5%, PPV of 96.9%, NPV of 25.0%, and diagnostic accuracy of 63.3%) [16].

Similar poor results of MASS were found in another study by Reyes-García et al. that applied the score for non-European populations at 2012, which reported area under the curve for MASS is 0.89. If surgical decision had been based on the modified Alvarado score, negative appendectomies would have been encountered in 18.3% of patients [17].

This study found that younger age, male gender, nausea/ vomiting and guarding were significant predictors for acute appendicitis according to modified RIPASA diagnosis in our study.

Reddy et al. reported that parameters like age, sex, duration of symptoms were also significant and have to be considered for diagnosis according to modified RIPASA [11].

### Study limitations

The design of the study which is cross sectional which has low precision and affect validity of results as there was difficulty in defining the time of onset of symptoms.

## Conclusion

The modified RIPASA score is the best diagnostic scoring system for acute appendicitis when compared to MASS, with the former achieving significantly higher sensitivity and diagnostic accuracy.

• Modified RIPASA was concluded to be a more applicable and useful score as it is non-invasive and reduces negative appendicectomy rates..

### Recommendations

From the study results we recommend:

1. Modified RIPASA score should be used as a tool that speeds up as well as enhances the accuracy of decision-making in cases of acute appendicitis.
2. Modified RIPASA lowers the need for expensive or potentially harmful investigations.

## Supplementary Information

**Additional file 1.**

## Data Availability

All date generated or analysed during this study are included in this published article and supplementary materials section.
